# Directed evolution induces tributyrin hydrolysis in a virulence factor of *Xylella fastidiosa* using a duplicated gene as a template

**DOI:** 10.12688/f1000research.5147.1

**Published:** 2014-09-09

**Authors:** Hossein Gouran, Sandeep Chakraborty, Basuthkar J. Rao, Bjarni Asgeirsson, Abhaya Dandekar

**Affiliations:** 1 Plant Sciences Department, University of California, Davis, CA, 95616, USA; 2 Department of Biological Sciences, Tata Institute of Fundamental Research, Homi Bhabha Road, Mumbai, 400 005, India; 3 Science Institute, Department of Biochemistry, University of Iceland, Dunhaga 3, IS-107 Reykjavik, Iceland

## Abstract

Duplication of genes is one of the preferred ways for natural selection to add advantageous functionality to the genome without having to reinvent the wheel with respect to catalytic efficiency and protein stability. The duplicated secretory virulence factors of *Xylella fastidiosa* (LesA, LesB and LesC), implicated in Pierce's disease of grape and citrus variegated chlorosis of citrus species, epitomizes the positive selection pressures exerted on advantageous genes in such pathogens. A deeper insight into the evolution of these lipases/esterases is essential to develop resistance mechanisms in transgenic plants. Directed evolution, an attempt to accelerate the evolutionary steps in the laboratory, is inherently simple when targeted for loss of function. A bigger challenge is to specify mutations that endow a new function, such as a lost functionality in a duplicated gene. Previously, we have proposed a method for enumerating candidates for mutations intended to transfer the functionality of one protein into another related protein based on the spatial and electrostatic properties of the active site residues (DECAAF). In the current work, we present *in vivo* validation of DECAAF by inducing tributyrin hydrolysis in LesB based on the active site similarity to LesA. The structures of these proteins have been modeled using RaptorX based on the closely related LipA protein from *Xanthomonas oryzae*. These mutations replicate the spatial and electrostatic conformation of LesA in the modeled structure of the mutant LesB as well, providing *in silico* validation before proceeding to the laborious *in vivo* work. Such focused mutations allows one to dissect the relevance of the duplicated genes in finer detail as compared to gene knockouts, since they do not interfere with other moonlighting functions, protein expression levels or protein-protein interaction.

## Introduction

The seminal and visionary work by Ohno in 1970 emphasized the pivotal role played by gene duplication in evolution. Gene duplication provides natural selection with the underlying mechanism to add functionality and adaptability to the genome by reusing pre-existing efficient and stable protein folds to catalyze novel reactions ^1^. The fate of duplicated genes - unchanged functionality, pseudogenization, subfunctionalization or neofunctionalization - is the focus of intense research enabled by the advancements in sequencing technologies ^2, 3^. Differing opinions on function innovation have also been articulated ^4^, and substantiated with real time evolution experiments ^5^.

Gene duplication plays a key role in the evolution of virulence-associated genes ^6^. Secreted lipases, one of the highly replicated genes in *Candida albicans* ^7^, has been implicated in disease pathogenesis, both in humans ^8^ and in plants ^9^. A lipase/esterase (LipA) from *X. oryzae* ( *Xanthomonas orysae pv. oryzaeraises* (Xoo)) that causes bacterial blight in rice and is conserved across the genus *Xanthomonas*, has been recently characterized ^10^. LipA also has three homologs (LesA, LesB and LesC) in the *Xylella fastidiosa* (Xf) genome ^11^.

Xf is a major source of concern for both economic ^12^ and food security reasons ^13^, being the causal agent for Pierce’s disease of grape (PD) and citrus variegated chlorosis (CVC) of citrus species ^14^. The presence of three duplicated genes (LesA/B/C) closely related to LipA from *X. oryzae* raises certain intriguing questions. It is logical to assume that these genes serve different purposes, since it is unlikely that three genes with identical functions will be maintained in the genome. A deeper understanding of their respective roles in the phenotypic context is essential in order to develop novel strategies to counter their threat ^15– 17^. LesA and LesB have 93% identity - yet, LesA can hydrolyze tributyrin whereas LesB can not. In the current work, we aim to induce tributyrin hydrolysis in LesB using minimal mutations.

The desire to mimic and accelerate natural evolution has fueled interest in directed evolution experiments, which endow or enhance functionality in enzymes. There has been some pioneering work in applying *de novo* methods to obtain catalytic functions ^18– 22^. However, most methods start with a template protein having the desired activity, known active site residues and 3D structure ^23– 25^. Previously, we have established a computational method (CLASP) based on spatial and electrostatic properties for the detection of active sites ^26– 29^, and a methodology to quantify promiscuity in proteins ^30^. We also explored the prospect of promiscuous active sites to serve as the starting point for directed evolution (DECAAF) ^31, 32^. DECAAF has been applied to the problem of identifying mutations in LesB based on the active site of LesA in order to endow LesB with tributyrin hydrolysis.

Since the structures of LesA/B are not known, and they share significant sequence homology with LipA (whose structure is known: PDBid:3H2G ^10^), we used RaptorX to model the LesA/B structures. We first verified that the electrostatic profile of LesA and LesB are different. The LesA and LesB structures were then superimposed, and residues within a radii of 6 Å (MUT1:three residues) and 8 Å (MUT2:eight residues, including the three residues in MUT1) from the residues of the catalytic triad in LesA were compared to those in LesB. The differing residues were identified as the set of mutations which would induce tributyrin hydrolysis in LesB. It was observed that MUT1 and MUT2 residues are in two different contiguous stretches in the protein. As a validation step, we modeled the mutated sequences of LesB using RaptorX, and analyzed the differences in their electrostatic profiles. We created two mutants for LesB: LesBMUT1 with three mutations, and LesBMUT2 with eight mutations. The mutations in LesBMUT1 replicated the electrostatic congruence in the MUT1 residues, but not in the MUT2 residues. Consequently, we expected LesBMUT2 to have tributyrin hydrolysis, but not LesBMUT1.

We tested for the activity of LesB wild type and LesBMUT1/LesBMUT2 proteins using two assays. In one we use agar plates containing emulsified tributyrin, which upon hydrolysis of tributyrin to glycerol and butyric acid makes a clear zone visible. The other assay is a fluorescent quantitative assay in which 4-methylumbelliferyl butyrate (4-MUB) is used as a fluorescent substrate. The tributyrin hydrolysis activity of wild type LesB and LesBMUT1/LesBMUT2 with suggested mutations was tested *in vitro* using heterologous expression of these proteins in *E. coli*. We were able to confirm the tributyrin hydrolysis activity of LesBMUT2 *in vitro* using the assays mentioned above, while LesBMUT1 showed no activity.

## Results and discussion

LipA and LesA/B/C multiple sequence alignment and 4-MUB assay data This zip file contains: a multiple sequence alignment for LipA and LesA/B/C (ALN format), and a CSV, which contains raw values for 4-MUB assay presenting relative fluorescent units (RFU). EV, empty vector; PBS, phosphate buffered saline.

**Figure 1.**
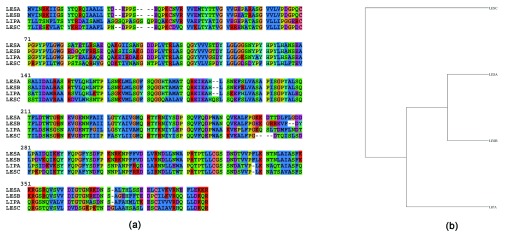
Multiple sequence alignment done using ClustalW, and phylogenetic trees generated using PhyML for LipA and LesA/B/C. ( **a**) Sequence alignment. ( **b**) Cladogram generated from ( **a**).

The sequence alignment for LipA ^10^, LesA (PD1703), LesB (PD1702) and LesC (P D1211) is shown in Figure 1a (gene names from http://www.ncbi.nlm.nih.gov/gene/). The phylogenetic tree and the pairwise sequence identity and similarity (Supplementary Table 1) suggests that LipA is more closely related to LesA/B than to LesC ( Figure 1b). For example, LesA and LesB have 90% identity (347 out of 387 residues are identical) and 93% similarity (361 out of 387 residues are similar). In lieu of these differences in LesB, it does not have the capability to hydrolyze tributyrin that LesA does.

**Figure 2.**
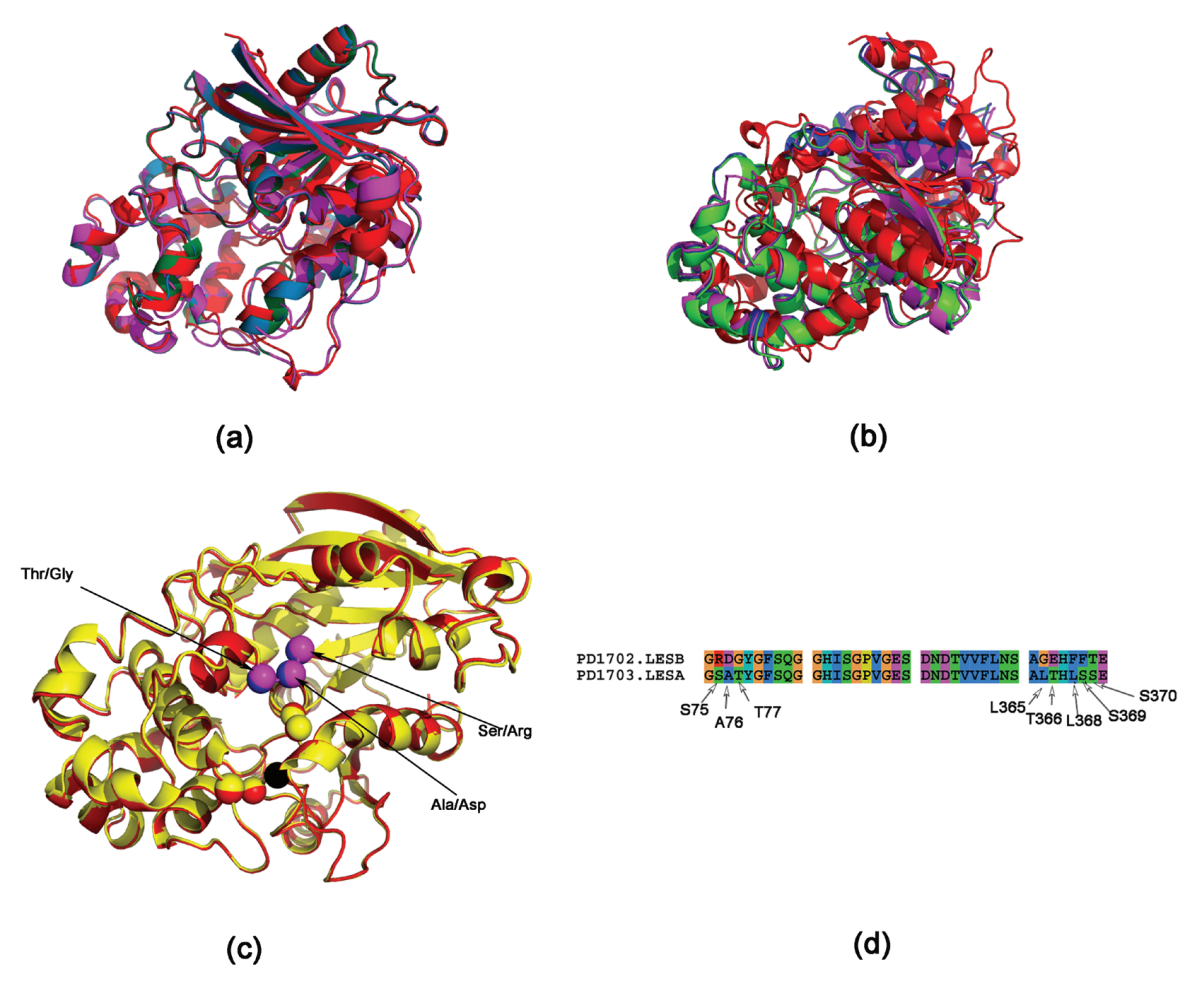
Superimposing LipA and LesA/B/C proteins. ( **a**) MUSTANG generated superimposition (LipA - red, LesA - green, LesB - blue, LesC - magenta). ( **b**) STEEP generated superimposition, obtained by superimposing three atoms from the catalytic triad in the active site. It can be seen that MUSTANG generates a better overall superimposition, since STEEP tries to get a better superimposition of the atoms, rather than achieve a global superimposition ^32^. ( **c**) Superimposition of LesA and LesB (red and yellow, respectively), obtained by superimposing the catalytic triad (Asp336, Ser176 and His377 in LipA). The Asp overlaps completely (at the origin of the coordinate system) and is shown in black. Some of the different residues are shown in magenta and blue for LesA and LesB respectively. ( **d**) Sequence alignment of active site residues (residues within a radii of 7 Å from the residues of the catalytic triad in LesA). Note that these residues are not in a one contiguous stretch.

The structures for LesA/B/C have not been solved. However, since there is significant sequence homology of these proteins with LipA, we modeled the structures of these proteins using RaptorX ^33^. RaptorX automatically chooses the best template (LesA - PDBid:3H2G, LesB and LesC - PDBid:3H2I) ^10^. The structural superimposition of these proteins done using MUSTANG ^34^ is shown in Figure 2a.

The active sites of these proteins are conserved and have a serine catalytic triad (Asp336, Ser176 and His377 in LipA). Previously, we have proposed a method to suggest mutations in a protein in order to endow it with a specific catalytic function based on structural and electrostatic homology of residues in the active site to a known protein with the desired function ^31^. We chose the C *β* atoms of the residues as the representative atom for each residue for an uniform comparison (only glycine lacks a C *β* atom). We have shown that although the reactive groups are different for amino acids, this difference is encapsulated in the backbone C *β* atoms ^35^. Table 1 shows the spatial and electrostatic potential difference (EPD) congruence in the catalytic triad in these proteins. We applied transformations to align the catalytic triad ( Figure 2b) ^31^.

**Table 1.** Potential and spatial congruence of the active site residues in LesA/B and the mutant LesB’s (LesBMUT1 and LesBMUT2). It can be seen that the serine catalytic triad is congruent in all proteins. Further, LesA and LesB have a different electrostatic profile when we consider another residue (Ser75/Arg75 respectively) - see pair ‘bd’ for instance. While the EPD in this pair is moderately positive in LesA (+38.5 EPD units), this is seen to change sign in LesB (-63.1 EPD units). The mutated structures (LesBMUT1 and LesBMUT2) modulates this EPD back to moderately positive again (+19 and +12 units in LesBMUT1 and LesBMUT2, respectively). D = Pairwise distance in Å. PD = Pairwise potential difference. See Methods section for units of potential.

PDB	Active site atoms (a,b,c,d)		ab	ac	ad	bc	bd	cd
LesA	ASP325CB,SER165CB, HIS367CB,SER75CB,	D PD	9.9 -61.9	4.9 -113.8	17.6 -23.5	7.0 -51.9	8.0 38.5	14.2 90.4
LesB	ASP323CB,SER165CB, HIS365CB,ARG75CB,	D PD	9.8 -48.8	5.1 -126.8	17.5 -111.9	6.8 -78.0	8.1 -63.1	14.0 14.9
LesBMUT1	ASP322CB,SER164CB, HIS364CB,SER74CB,	D PD	9.8 -90.3	4.9 -118.2	17.5 -71.0	6.8 -27.9	8.1 19.3	14.0 47.2
LesBMUT2	ASP323CB,SER165CB, HIS365CB,SER75CB,	D PD	9.9 -69.4	5.1 -114.1	17.7 -57.0	6.9 -44.7	8.2 12.4	14.2 57.1

This multiple superimposition of the proteins provided a single frame of reference for comparing the proteins LesA and LesB ( Figure 2c). After the superimposition, we took residues within a radii of 7 Å from the residues of the catalytic triad in LesA. Now, for each of these residues we found the closest residue in LesB - noting that they are now superimposed. The different residues, that form the set which are to be mutated, are shown in Table 2 and Figure 2d. It can be seen that these residues lie within two contiguous stretches: a) R75, D76 and G77 (MUT1) which are about 6 Å away from the active site residues and b) G363, E364, F366, F367 and T368 (MUT2) that are about 8 Å away from the active site residues. We created a LesB mutant by mutating resides in (a) (LesBMUT1), and another (LesBMUT2) by mutating residues in both sets.

**Table 2.** Mutations required to mimic the LesA active site in the LesB protein. Two sets of mutations were studies here - LesBMUT1 and LesBMUT2. The LesBMUT1 mutations are within 6 Å of the active site residues, and located within a contiguous stretch. We further added the LesBMUT1 mutations to another set of residues that differ (8 Å away from the active site residues) to obtain LesBMUT2 mutations.

LesB	LesA	LesBMUT1	LesBMUT2
R75	S75	S75	S75
D76	A76	A76	A76
G77	T77	T77	T77
G363	L365	-	L363
E364	T366	-	T364
F366	L368	-	L366
F367	S369	-	S367
T368	S370	-	S378

As a validation step, we modeled the structures of the mutated sequences in order to compare the change in the electrostatic profile. We first consider the catalytic triad and one of the mutated amino acid (Arg75 to Ser75 in LesB) in the LesBMUT1/LesBMUT2 structures ( Table 1). It can be seen that the mutated active site is spatially similar to wild type. The same holds true for all structures discussed henceforth. The major change in the electrostatic profile can be seen for the pair ‘bd’ (Ser165CB/Arg75CB in LesB versus Ser165CB/Ser75CB in LesBMUT1/LesBMUT12). While the EPD in this pair is moderately positive in LesA (+38.5 EPD units), this is seen to change sign in LesB (-63.1 EPD units). The mutated structures (LesBMUT1/LesBMUT2) modulate this EPD back to moderately positive again (+12.4 EPD units).

While it may appear from Table 1 that the three mutations (MUT1) in LesBMUT1 might suffice, it is necessary to compute the changes in the MUT2 residues. We chose one residue from the active site (Asp323) and three residues from the MUT2 set (E364, F366 and F367). Since Gly does not have a C *β* atom, it could not be used for comparison. There are certain differences in the electrostatic profile between LesBMUT1 and LesBMUT2. While the EPD in ‘bd’ is moderately positive in LesA (+8.2 EPD units), this changes sign in LesBMUT1 (-72.3 EPD units) ( Table 3). However, this EPD modulates back to moderately positive again in LesBMUT2. Thus, it might be necessary to introduce all eight mutations (MUT1 + MUT2) in order to introduce tributyrin hydrolysis in LesB. This verification step is unique to our methodology, and infuses confidence in the chances of success with the *in vivo* mutations. We now made two mutants of LesB - LesBMUT1 having three mutations, and LesBMUT2 having eight mutations.

**Table 3.** Potential and spatial congruence of the active site residues in LesA and the two LesB variants (LesBMUT1 and LesBMUT2). We chose one residue from the active site (Asp323) and three residues from the MUT2 set (E364, F366 and F367). Since Gly does not have a C *β* atom, it could not be used for comparison. We observed certain differences in the electrostatic profile between LesBMUT1 and LesBMUT2 - see pair ‘bd’ for instance. While the EPD in this pair is moderately positive in LesA (+8.2 EPD units), this is seen to change sign in LesBMUT1 (-72.3 EPD units). This EPD modulates back to moderately positive again in LesBMUT2. Thus, it might be necessary to introduce all eight mutations (MUT1 + MUT2) in order to introduce tributryn catalysis in LesB. D = Pairwise distance in Å. PD = Pairwise potential difference. See Methods section for units of potential.

PDB	Active site atoms (a,b,c,d)		ab	ac	ad	bc	bd	cd
LESA	ASP325CB,THR366CB, LEU368CB,SER369CB,	D PD	5.2 -20.8	9.4 -38.6	8.4 -12.6	7.2 -17.8	5.2 8.2	5.4 26.0
LESBMUT1	ASP322CB,GLU363CB, PHE365CB,PHE366CB,	D PD	4.8 -8.6	9.3 -118.2	8.4 -80.9	7.5 -109.6	5.5 -72.3	5.5 37.3
LESBMUT2	ASP323CB,THR364CB, LEU366CB,SER367CB,	D PD	4.9 -13.4	9.5 -44.1	8.5 5.4	7.5 -30.7	5.4 18.9	5.4 49.6

**Figure 3.**
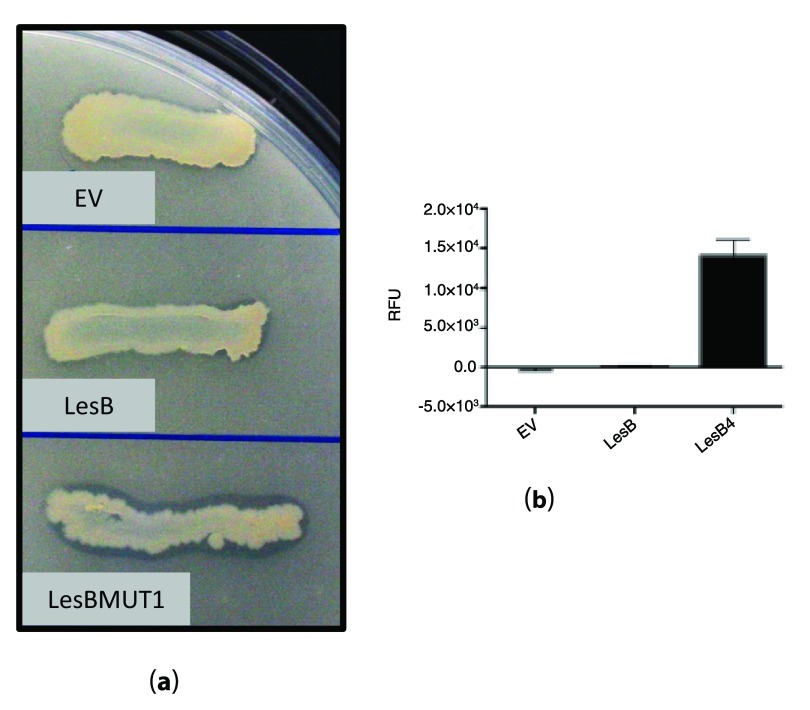
Assays to detect tributyrin hydrolysis activity for LesB, LesBMUT2 and EV (empty vector). ( **a**) Tributyrin agar plate assay. Tributyrin hydrolysis zone (clearing zone) is visible for *E. coli* strains expressing LesBMUT2 and not for LesB or empty vector (EV) strains. ( **b**) A4-Methylumbelliferyl butyrate (4-MUB) fluorescent assay detecting tributyrin hydrolysis activity of LesB, LesBMUT2 and EV (empty vector) proteins expressed in *E. coli*.

To test the activity of LesBMUT1/LesBMUT2 *in vitro*, we expressed LesB and LesBMUT1/LesBMUT2 in *E. coli* using an expression vector to obtain high quantities of heterologous protein. The *E. coli* strain expressing LesBMUT2 streaked on tributyrin agar plates showed significant tributyrin hydrolysis activity compared to wild type LesB. The activity was visualized by a tributyrin hydrolysis zone on the agar plate containing emulsified tributyrin fat ( Figure 3a). However, the LesBMUT1 showed no tributyrin hydrolysis under the same conditions.

Using 4-MUB assay we were able to detect tributyrin hydrolysis activity and confirm what we observed in tributyrin agar plate. Crude protein extracted from LesBMUT2 expressing *E. coli* showed a significant increase in tributyrin hydrolysis activity as depicted in Figure 3b.

Asn228 in LipA is an interesting residue in the context of tributyrin hydrolysis. This residue is involved in the binding of glycoside detergent *β*-octyl glucoside (BOG) in LipA (PDBid:3H2K) in *X. oryzae* pv oryzae. Interestingly, while the mutant N228W mutant (PDBid:3H2I) is still able to hydrolyze tributyrin, it showed virulence deficiency similar to that of the LipA-deficient strain BXO2001 ^36^. Corroborating the redundancy of Asn228 in tributyrin hydrolysis, we note that this residue falls outside the 8 Å radius chosen for LesBMUT2 mutations, which were able to induce tributyrin hydrolysis.

## Materials and methods

### Heterologous protein expression in *E. coli*

In order to test for tributyrin hydrolysis activity, the new open reading frame with suggested mutations as well as wild type LesB was codon optimized for expression in *E. coli* and were chemically synthesized (DNA2.0, Menlo Park, CA). The resulting coding sequences were cloned into pJ401: T5 expression vector from DNA2.0 to obtain high amounts of recombinant LesB4 and LesB.

### Cell culture and protein extraction

*E. coli* strains expressing LesB wild type and LesB4 as well as empty vector control (EV) were inoculated into liquid cultures and grown overnight at 37°C with constant shaking at 200 rpm. The overnight cultures were added to a larger flask with fresh LB media and grown under the same conditions until they reached OD of 0.5–0.8, at the point which 0.3 mM of IPTG (Sigma Aldrich) was added to each culture to induce the promoter. Induced cultures were incubated at room temperature with constant shaking at 200 RPM overnight. The following day cultures were spun down at 5000g (Sigma 3K10) to pellet the cells and resulting pellet was suspended in sterile PBS. Next, the obtained cells in PBS were lysed using a microfluidizer (Microfluidics M-110L) machine. The extracted protein was quantified and used for the 4-MUB assay.

### 4-Methylumbelliferyl butyrate (4-MUB) assay

The quantitative detection of LesA enzyme was carried out using MUB Assay based on Vaneechoutte *et al.* ^37^. For each samples 3 technical replicates were used. Briefly, A stock solution of the substrate, made by dissolving 10 mg of 4-methylumbelliferyl butyrate (4-MUB) (Sigma Chemical Co., St. Louis, MO) in 1 mL of dimethyl sulfoxide (DMSO) and 10 μL of Triton X-100 to obtain 40 mM stock solution which was further diluted to 5 mM using 0.1 M citrate buffer (pH 5.0). For each reaction 80 μL of substrate was added to 20 μL of each sample immediately before the fluorescent intensity was read in a fluorometer Plate Reader SpectraMax M2 (Molecular Devices) at 365 nm excitation and 455 nm emission at 30°C for 30 minutes. Fluorescence values measured in 4-MUB assay were used to calculate mean and standard deviations. After subtracting the background (PBS) values were plotted using Prism version 6.0c.

***In silico methods.*** The current work provides *in vivo* validation of previously described *in silico* methods ^26, 31, 32^. The underlying theoretical foundation for our methods is the non-triviality of the spatial and electrostatic congruence in cognate pairs seen across various structures of the same catalytic function. We identify spatially equivalent residues that have differing electrostatic properties, based on the logic that functional divergence in the protein family arises from these residues.

In order to superimpose the two scaffolds - LesA=(Asp336, Ser176 and His377) and LesB=(Asp323, Ser165 and His365) - we applied linear and rotational transformations for all atoms in LesA and LesB such that the C *β* atoms of all residues lay on the same plane (Z=0), ${\text{Asp336}}_{\text{LesA}}^{C\beta }$ and ${\text{Asp323}}_{\text{LesB}}^{C\beta }$ were at the center, and ${\text{Ser176}}_{\text{LesA}}^{C\beta }$ and ${\text{Ser165}}_{\text{LesB}}^{C\beta }$ lie on the Y axis. This superimposition was outputted as a Pymol formatted file.

 We aligned the residues from LesA which are within 7 Å radius from the active site residues (Asp336, Ser176 and His377). The choice of the radial distance that encompasses interacting residues is critical, since a small radius will not include enough residues, and a large one will include irrelevant ones. Next, for LesB we identified residues that were in the vicinity of each of the residues in LesA, choosing the closest residue as the alignment for the position *p*. This is possible since we have a consolidated spatial reference frame for both the proteins. RaptorX was then used to predict the structure of the mutated LesB ^33^.

Adaptive Poisson-Boltzmann Solver (APBS) and PDB2PQR packages were used to calculate the potential difference between the reactive atoms of the corresponding proteins ^38, 39^. The APBS parameters and electrostatic potential units were set as described previously in ^26^.

All protein structures were rendered by PyMol ( http://www.pymol.org/). The alignment and cladograms images were created using Seaview ^40^. PHYML was used to generate phylogenetic trees from these alignments, which searches for a tree with the highest probability or likelihood that, given a proposed model of evolution and the hypothesized history, would give rise to the observed data set (method of maximum likelihood) ^41^.

## Data availability

F1000Research: Dataset 1. LipA and LesA/B/C multiple sequence alignment and 4-MUB assay data, 10.5256/f1000research.5147.d34954 ^42^
