# Supplementary material for: Directed evolution induces tributyrin hydrolysis in a virulence factor of Xylella fastidiosa using a duplicated gene as a template
Source: F1000Res. 2014 Sep 9;3:215. [Version 1] doi: 10.12688/f1000research.5147.1 (PMC4329599; doi:10.12688/f1000research.5147.1)
Supplement: Peer review history

# Peer review: Referee response for version 1

**DOI:** 10.5256/f1000research.5490.r7453

**Published:** 2015-02-16

**Authors:** Tomomichi Fujita

**Affiliations:** 1 Faculty of Science, Hokkaido University, Sapporo, Japan

In this article, the authors clearly demonstrate usefulness of their *in silico* methods to identify residues which are spatially equivalent but electrostatically different in cognate pairs in protein family, then provide *in vivo* validation of the method by measuring the enzymatic activity of the designed protein. Such “focused mutation” approach and the experimental validation allow us to identify minimal mutations, which are important for the enzymatic activity and also may shed light on the fate of, for example, the duplicated gene products; pseudogenization, subfunctionalization or neofunctionalization.

**Minor comments for improvement of article.**

The authors aimed to induce tributyrin hydrolysis in LesB using minimal mutations. They described either three (MUT1) or eight mutations (MUT2) in LesB protein, where MUT1 did not show tributyrin hydrolysis activity, but MUT2 did. However, I wonder if the number of mutations, eight could be minimal mutations or not. If the authors have other mutations introduced, such as between four and seven mutations in LesB, please explain them.

In Fig. 1(a), amino acid positions are numbered, however, to which protein are the numbers? Also, please mark three active site residues of LipA (serine catalytic triad) in the figure.

In Fig. 2(c), please indicate five amino acid residues (G363, E364, F366, F367 and T368) in another contiguous stretch. Also, please show the catalytic triad as well.

In Table 1, please indicate to which amino acid residues “a, b, c, d” of active site atoms correspond, respectively.

In Table 1, I don’t understand amino acid positions, SER 164, HIS 364, and SER 74 in LesBMUT1, which are different from the positions, SER 165, HIS 365, and ARG/SER 75 in LesB/LesBMUT1, respectively.

In text, page 5, a right column, line 18 from bottom, “…(LesBMUT1/LesBMUT2) modulate this EPD back to moderately positive again (+12.4 EPD units).” would be “…(LesBMUT1/LesBMUT2) modulate this EPD back to moderately positive again (+19.3 and +12.4 EPD units, respectively).”

In text, page 6, a left column, it would be better to show the results of LesBMUT1, showing no hydrolysis activity in Fig. 3(a), (b). If the authors have the data of LesA, please show them together in Fig. 3(a), (b).

In Fig. 3(a), LesBMUT1 should be LesBMUT2.

In Fig. 3(b) and in Materials and methods, please explain what LesB4 is.

In Table 3, whereas the EPD in the pair ‘ad’ of LESA and LESBMUT1 are negative, the EPD in the one of LESBMUT2 is positive (+5.4), showing inconsistency of the presence of the enzymatic activity in LESA and in LESBMUT2, but not in LESBMUT1. Please explain this.

In Materials and methods, cell culture and protein extraction, the extracted protein was quantified and used for the 4-MUB assay. Please describe how the authors normalized protein concentration in each sample to calculate mean and standard deviation values for the 4-MUB assay.

In Materials and methods, a right column, line 14, what does ‘the position p’ mean, which is described as in “the alignment for the position p”?

I have read this submission. I believe that I have an appropriate level of expertise to confirm that it is of an acceptable scientific standard.

# Peer review: Referee response for version 1

**DOI:** 10.5256/f1000research.5490.r6084

**Published:** 2014-09-24

**Authors:** Rajan Sankaranarayanan

**Affiliations:** 1 Structural Biology Laboratory, Centre for Cellular and Molecular Biology, Hyderabad, India

In this study, the authors have modeled the duplicated secreted virulence factors of *Xylella fastidiosa* (LesA, LesB, LesC), based on the closely related LipA protein structure from *Xanthomonas oryzae*. LesA protein can hydrolyze tributyrin while LesB and LesC cannot, although LesB and LesC both have a catalytic triad (Asp, Ser, His) similar to LesA and LipA. 

The authors have used a previously developed computational method by their group that suggests mutations based on the spatial and electrostatic properties of the active site residues. In this study, LesA active site was used to suggest mutations in LesB. After the introduction of these mutations, LesB shows activity upon tributyrin. This piece of work is a good example theoretically predicting functionality that can narrow down the search in the sequence space.

**Comments/ Suggestions:**

1. Were tributyrin hydrolysis assays done for LipA and LesA? A comparison would portray the differential effect of mutations. 
2. In figure 3(a), the third image was mislabeled as LesBMUT1 instead of LesBMUT2.
3. What does DECAAF stands for? How the spatially equivalent residues were identified which differ in their electrostatic properties? A brief explanation about how this algorithm works would have been better to appreciate the work.
4. The title needs to be changed as it conceptually clashes with 'Directed evolution' used in common parlance, which is experiment based but a black box methodology. Here, they use certain available structural information and hence should not be confused with the above term.

I have read this submission. I believe that I have an appropriate level of expertise to confirm that it is of an acceptable scientific standard.
